# The effects of naltrexone on retention in treatment and being opioid-free in opioid-dependent people: A systematic review and meta-analysis

**DOI:** 10.3389/fpsyt.2022.1003257

**Published:** 2022-09-26

**Authors:** Moein Zangiabadian, Saeid Golmohammadi, Seyed Aria Nejadghaderi, Mohammad Mahdi Zahmatkesh, Mohammad Javad Nasiri, Majid Sadeghian

**Affiliations:** ^1^Department of Microbiology, School of Medicine, Shahid Beheshti University of Medical Sciences, Tehran, Iran; ^2^Department of Psychiatry, Faculty of Medicine, Shahid Beheshti University of Medical Sciences, Tehran, Iran

**Keywords:** opioid, naltrexone, retention in treatment, opioid free, systematic review, meta-analysis

## Abstract

**Background:**

Opioid dependency is a chronic relapsing disorder for which different therapeutically interventions have been developed. Naltrexone is a non-selective opioid antagonist that can be utilized for maintenance therapy in opioid dependency. In this systematic review, we aimed to evaluate the effects of naltrexone on retention in treatment and being opioid-free.

**Methods:**

We systematically searched PubMed and EMBASE databases up to February 5, 2022, using the following keywords: “Naltrexone,” “Substance abuse,” “Drug abuse,” “Opiate-related disorder,” and “Opioid dependence.” Studies that included opiate-dependent individuals who were treated with naltrexone and assessed retention in treatment or being opioid-free were included. Two authors independently used the Cochrane risk-of-bias tool for quality assessment. A random effect model in Comprehensive Meta-Analysis software was used for the conduction of the meta-analysis. We performed subgroup analysis to evaluate the effects of naltrexone types on outcomes.

**Results:**

Eighteen studies, including 2,280 participants met our inclusion criteria. The duration of treatment ranged from 21 days to 24 months. The retention in treatment with naltrexone was 63% higher than controls (odds ratio (OR): 1.64 [95% confidence interval (CI), 0.78–3.44]. The OR for being opioid-free was 1.63 (95% CI, 0.57–4.72). Injectable naltrexone was significantly effective on retention in treatment (OR 1.86; 95% CI, 1.17–2.98).

**Conclusions:**

We found that naltrexone could be useful for retention in treatment and being opioid-free, however, the findings were not significant. Further high-quality and large-scale observational studies are recommended.

## Introduction

During the past decade, the number of opioid users for non-medical purposes has increased and achieved ~62 million people around the world (about 1.2 percent of the worldwide population). Also, about 50,000 people died in 2019 in the United States due to opioid overdose which has doubled since 2010 (1). Opioid dependency is a chronic relapsing disorder. Long-term opioid exposure causes up-regulation of the cyclic adenosine monophosphate pathway mediated by adenylyl cyclase. This is one of the mechanisms that will lead to symptoms of opioid withdrawal (2). Individuals with opioid dependency have an increased risk for health-related disorders, including injuries, suicide, human immunodeficiency virus and hepatitis C virus infections, and bacterial endocarditis (3). In addition to personal harm, a wide range of social impacts are associated with opioid dependencies such as crime and violence growth, damage to family relations, reduced job opportunities, and economic cooperation (3, 4). Opioid withdrawal has a group of behavioral, physiological, and cognitive symptoms such as sweating, anxiety, insomnia, anhedonia, and hyperalgesia so appropriate interventions is important for compliance with treatment in opioid-dependent patients (5, 6).

Pharmacological and non-pharmacological interventions such as psychological counseling and group meeting are used for opioid dependency treatment (7). According to the American Psychiatric Association (APA) guideline for pharmacological opioid dependency management; treatment commonly is initiated with acute detoxification and then is continued by long-term opioid replacement therapy (8). There are several types of long-term pharmacological therapies such as agonists, partial agonists, and antagonists of opioid receptors, like treatment with methadone, buprenorphine, and naltrexone, respectively. Also, combined therapies like buprenorphine/naloxone can be used (7, 9).

Naltrexone is a non-selective opioid antagonist that can be utilized for maintenance therapy in opioid dependency. It is a long-acting pure antagonist with a high affinity to μ receptors. Six-beta-naltrexone is the main and long-acting metabolite of naltrexone, which prolong the antagonistic effects of naltrexone on narcotic receptors (10, 11).

A previous systematic review has evaluated the efficacy, adherence, and overdose rates of extended-release naltrexone for opioid use disorders (12). Meta-analysis was not performed in that study. Moreover, the study only included studies up to June 2017 (12). Herein, we aimed to assess the efficacy (being opioid-free) and compliance (retention in treatment) of naltrexone on individuals with opioid dependency.

## Methods

### Search strategy

We searched PubMed and EMBASE for studies reporting the therapeutic effects of naltrexone on opioid-dependent patients, published up to February 5, 2022. The search terms were as follow: {[Naltrexone (MeSH Terms)] OR [Naltrexone (Title/Abstract)]} AND {[substance abuse (Title/Abstract) OR drug abuse (Title/Abstract) OR opiate-related disorder (Title/Abstract) OR opioid dependence (Title/Abstract)] OR [Opioid-Related Disorders (MeSH Terms)]}. Only clinical trials written in English were selected. Backward and forward citation searching was performed. This study was conducted and reported by the Preferred Reporting Items for Systematic Reviews and Meta-Analyses (PRISMA) 2020 statement (13).

### Study selection

The records found through database searching were imported and deduplicated using EndNote X8 (Thomson Reuters, Toronto, ON, Canada). Two reviewers independently screened the records by title/abstract and full text to exclude those unrelated to the study objectives. Included studies met the following criteria: (1) opiate-dependent patients; (2) patients treated with naltrexone; (3) treatment success (retention in treatment or being opioid-free at the end of the study without any relapse). Studies that discuss the effect of other drugs like nalmefene or survey the therapeutic effect of naltrexone on non-dependent or alcohol-dependent patients or focus on early detoxification were excluded.

### Data extraction

Two reviewers designed a data extraction form and extracted data from all eligible studies, with differences being resolved by consensus. The following data were extracted: first author's name; year of publication; country or countries where the study was conducted; patient age; treatment protocols (treatment regimens and duration of treatment); the definition of case and control; the total number of controls and cases and the definition of treatment success.

### Quality assessment

Two reviewers assessed the quality of the studies using the Cochrane tool for experimental studies (14). A third reviewer was involved in case of inconsistencies. The Cochrane tool is based on; the use of random sequence generation; concealment of allocation to conditions; blinding of participants and personnel; blinding of outcome assessors; completeness of outcome data and other; selective reporting and other biases. Each study was rated as at low risk of bias when there was no concern regarding bias; as high risk of bias when there was concern regarding bias; or at unclear risk of bias if the information was absent.

### Statistical analysis

The pooled odds ratios (ORs) with a 95% confidence interval (CI) were assessed using random or fixed-effect models. The random-effects model was used in treatment outcome analysis because of the estimated heterogeneity of the true effect sizes and both random and fixed-effect models were used in subgroup analysis. Moreover, subgroup analysis was performed to compare the effect of naltrexone type on each outcome. The between-study heterogeneity was assessed by Cochran's Q and the *I*^2^ statistic. *I*^2^ values of more than 50% were considered as high heterogeneity (15). Publication bias was evaluated statistically by using Egger's and Begg's tests (*p* < 0.05 was considered indicative of statistically significant publication bias) (16). All analyses were performed using “Comprehensive Meta-Analysis” software, Version 2.0 (Biostat, Englewood, NJ).

## Results

**Figure 1:**
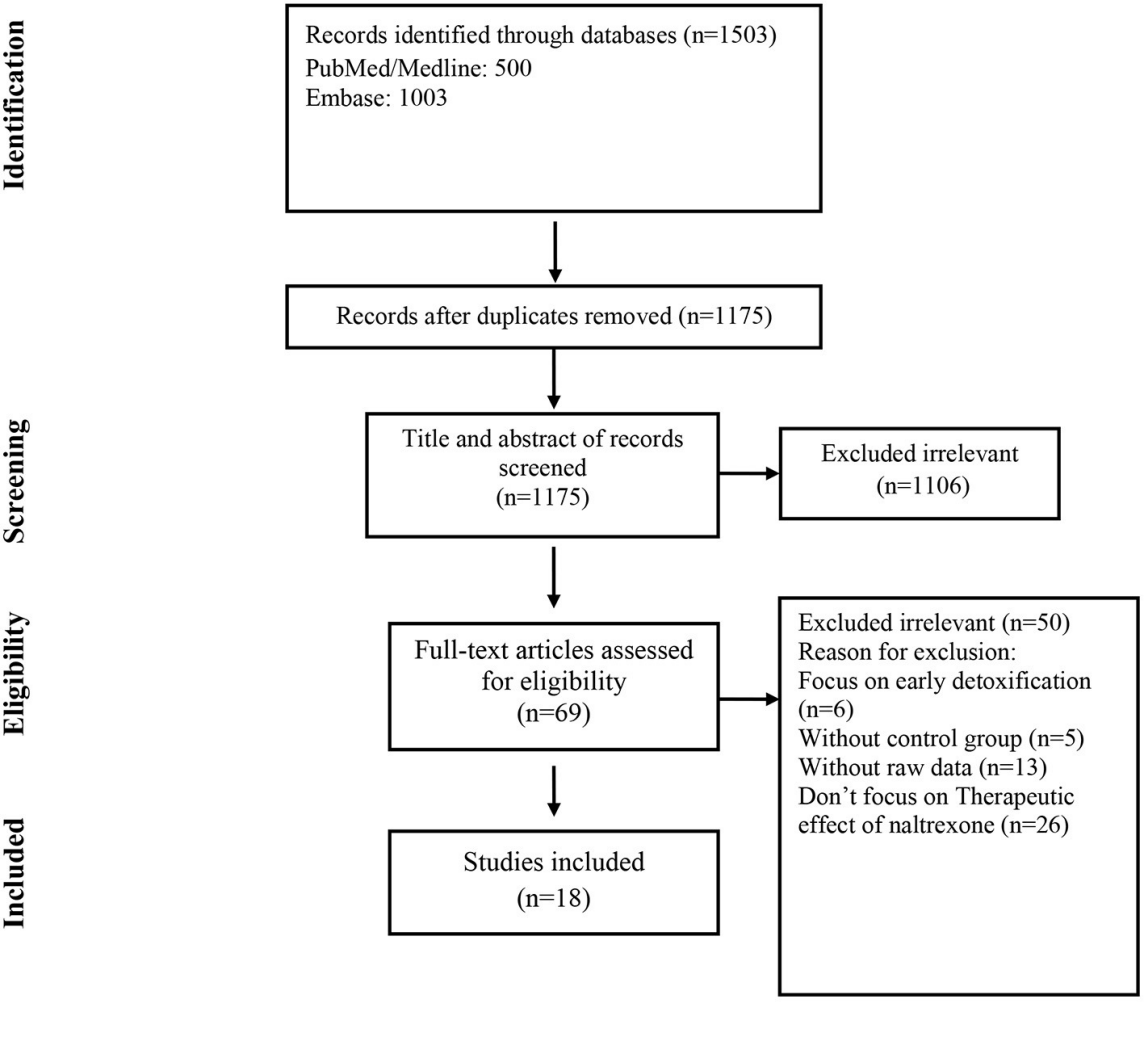
Flow chart of study selection for inclusion in the systematic review and meta-analysis.

The selection process of articles is shown in Figure 1. Eighteen articles were included in (17–34). There were 1,105 cases and 1,175 controls in studies with a total population of 2,280 participants. According to thirteen studies (17, 19–25, 27, 28, 30–32), the mean age of both cases and controls was 34 years, respectively, also the total mean age of all participants was 34 years according to fifteen studies (17–25, 27, 28, 30–32, 34). Seven studies were conducted in the USA (17–19, 24, 25, 27, 34), three in Russia (21, 22, 28), two in Malaysia (29, 31), and Israel (26, 32) and one in Norway, Germany, Italy and Spain (20, 23, 30, 33). The characteristics of the included articles are summarized in Table 1.

**Table 1:** Included study characteristics.

**References**	**Study design**	**Published year**	**Country**	**Age (mean)**		**Definition of case (number of participants)**	**Definition of control (number of participants)**
				**Case**	**Control**		
San et al. (30)	Double blind randomized controlled clinical trial	1991	Spain	26.1	22	Treatment by naltrexone after detoxification and 1 month administration of naltrexone (23)	Treatment by placebo after detoxification and 1 month administration of naltrexone (20)
Lerner et al. (26)	Double blind clinical trial	1992	Israel	NR		Receiving naltrexone (15)	Receiving placebo (16)
Shufman et al. (32)	Double blind randomized clinical trial	1994	Israel	33.6	32	Treatment by naltrexone after detoxification (16)	Treatment by placebo after detoxification (16)
Cornish et al. (18)	Randomized clinical trial	1997	USA	39		Naltrexone and brief drug counseling (34)	Counseling alone (17)
Stella et al. (33)	Randomized clinical trial	2005	Italy	NR		Patients receiving naltrexone (14)	Patients receiving psychological support (not pharmacological treatment) (14)
Grusser et al. (20)	Non-randomized clinical trial	2006	Germany	30.6	34.7	Patients received a Naltrexone after detoxification (17)	Patients receiving Levomethadone after detoxification (17)
Comer et al. (17)	Double blind randomized clinical trial	2006	USA	41	40	Long acting naltrexone administered (22)	Placebo administered (18)
Manneilli et al. (27)	Open-label naturalistic trial	2007	USA	32.9	32.7	Naltrexone + clonidine + psychosocial treatment (162)	Clonidine + psychosocial treatment (273)
Schottenfeld et al. (31)	Double blind randomized clinical trial	2008	Malaysia	38.2	37.6	Assigned to naltrexone +drug counseling (29)	Assigned to placebo +drug counseling (23)
Kunoe et al. (23)	Randomized, open-label, trickle-inclusion trial	2009	Norway	34.5	34	Naltrexone administration after pass an 25 mg oral naltrexone challenge (27)	Usual care (vocational counseling, readmission to detoxification) (29)
Coviello et al. (19)	Randomized clinical trial	2010	USA	33.1	33.9	Naltrexone + Psychosocial Treatment (56)	Psychosocial Treatment (55)
Krupitsky et al. (21)	Double-blind randomized clinical trial	2011	Russia	29.4	29.7	Extended release naltrexone + counseling (126)	Placebo + counseling (124)
Krupitsky et al. (22) Group 1	Double-blind, double-dummy, randomized clinical trial	2012	Russia	27.9	28.7	Naltrexone implant + oral placebo + counseling (102)	Placebo implant and oral placebo + counseling (102)
Krupitsky et al. (22) Group 2	Double-blind, double-dummy, randomized clinical trial	2012	Russia	28	28.7	Placebo implant + oral naltrexone hydrochloride + counseling (102)	Placebo implant and oral placebo + counseling (102)
Ruger et al. (29)	Double-blind randomized clinical trial	2012	Malaysia	NR		Naltrexone + counseling (43)	Placebo + counseling (36)
Sullivan et al. (34)	Double-blind clinical trial	2013	USA	41		Receiving naltrexone (21)	Receiving placebo (17)
Lee et al. (25)	Open-label, non-blinded, non-placebo randomized clinical trial	2015	USA	40	47	Extended-release naltrexone administered (17)	Usual care (no medication treatment-as-usual) (17)
Lee et al. (24)	Open-label, randomized clinical trial	2016	USA	44.4	43.2	Extended-release naltrexone administered (153)	Usual treatment (brief counseling and referrals for community treatment programs) (155)
Nunes et al. (28)	Double-blind, randomized clinical trial	2019	Russia	29.4	29.7	Extended-release naltrexone administered (126)	Placebo administered (124)

NR, not reported.

### Quality of the included studies

Based on the Cochrane tool which was used to evaluate the quality of the clinical trials, five studies had a low risk of bias (19, 21, 22, 26, 31). Five studies had bias only in the cases of assessor blinding (28, 29, 34) and allocation concealment (30, 32) and three studies had a high risk of bias only in the cases of blinding of participants, and blinding of outcome (23–25). Other studies had a high risk of bias for randomization, group concealment, participant assignment, and assessor blinding (18, 20, 27, 33) (Table 2).

**Table 2:** Quality assessment of the experimental studies included in the meta-analysis using the Cochrane risk of the bias assessment tool.

**References**	**Random sequence generation**	**Allocation concealment**	**Blinding of participants and personnel**	**Blinding of outcome assessment**	**Incomplete outcome data**	**Selective reporting**	**Other bias**
Schottenfeld et al. (31)	Low risk	Low risk	Low risk	Low risk	Low risk	Low risk	Low risk
Manneilli et al. (27)	High risk	High risk	High risk	High risk	Low risk	Low risk	Low risk
Comer et al. (17)	Low risk	High risk	Low risk	Low risk	Low risk	Low risk	Low risk
Grusser et al. (20)	High risk	High risk	High risk	High risk	Low risk	Low risk	Low risk
Stella et al. (33)	Low risk	High risk	High risk	High risk	Low risk	Low risk	Low risk
Cornish et al. (18)	Low risk	High risk	High risk	High risk	Low risk	Low risk	Low risk
Shufman et al. (32)	Low risk	High risk	Low risk	Low risk	Low risk	Low risk	Low risk
Lerner et al. (26)	Low risk	Low risk	Low risk	Low risk	Low risk	Low risk	Low risk
San et al. (30)	Low risk	High risk	Low risk	Low risk	Low risk	Low risk	Low risk
Coviello et al. (19)	Low risk	Low risk	Low risk	Low risk	Low risk	Low risk	Low risk
Krupitsky et al. (21)	Low risk	Low risk	Low risk	Low risk	Low risk	Low risk	Low risk
Krupitsky et al. (22)	Low risk	Low risk	Low risk	Low risk	Low risk	Low risk	Low risk
Kunoe et al. (23)	Low risk	Low risk	High risk	High risk	Low risk	Low risk	Low risk
Lee et al. (25)	Low risk	Low risk	High risk	High risk	Low risk	Low risk	Low risk
Lee et al. (24)	Low risk	Low risk	High risk	High risk	Low risk	Low risk	Low risk
Nunes et al. (28)	Low risk	Low risk	Low risk	High risk	Low risk	Low risk	Low risk
Ruger et al. (29)	Low risk	Low risk	Low risk	High risk	Low risk	Low risk	Low risk
Sullivan et al. (34)	Low risk	Low risk	Low risk	High risk	Low risk	Low risk	Low risk

### Intervention characteristics

The duration of the intervention range was from 21 days to 24 months. Oral and injectable or implant naltrexone had been administered in nine (18, 19, 26, 27, 29–33) and eight (17, 20, 21, 23–25, 28, 34) studies, respectively. Krupitsky et al. (22) compared the effect of oral and implant naltrexone in two different groups. The duration, type, and dosage of intervention are shown in Table 3.

**Table 3:** Intervention characteristics.

**References**	**Type of intervention**	**Duration of intervention**	**Dosage of intervention**
San et al. (30)	Oral treatment by naltrexone	6 months	NR
Lerner et al. (26)	Oral treatment by naltrexone	2 months	NR
Shufman et al. (32)	Oral treatment by naltrexone	3 months	50 mg per week for first 2 weeks followed by 150 mg per week
Cornish et al. (18)	Oral treatment by naltrexone	6 months	25 mg daily for 2 days, followed by 50 mg daily for 3 days. Approximately 1 week after initiation, subjects were stabilized on a naltrexone regimen of 100 mg on Tuesdays and 150 mg on Fridays.
Stella et al. (33)	Oral treatment by naltrexone	6 months	50 mg naltrexone daily
Grusser et al. (20)	Depot-Naltrexone-Pellet administration	6 weeks	NR
Comer et al. (17)	Injectable depot long acting naltrexone administered	2 months	384 mg
Manneilli et al. (27)	Oral treatment by naltrexone	21 days	The naltrexone dose escalation schedule was as follows: Day 1, 1 mg; Day 2, 2 mg; Day 3, 3 mg; Days 4–6.5 mg; Days 7–21.10 mg
Schottenfeld et al. (31)	Oral treatment by naltrexone	6 months	One 50-mg tablet of naltrexone were given every day during the first week of maintenance. Subsequently, patients received two 50-mg tablets of naltrexone every Monday and Wednesday, and three50-mg tablets of naltrexone every Friday
Kunoe et al. (23)	Naltrexone implant	6 months	20 pellets containing ~2.2 g naltrexone implant
Coviello et al. (19)	Oral treatment by naltrexone	6 months	300 mg per week
Krupitsky et al. (21)	Injectable extended release naltrexone	6 months	380 mg
Krupitsky et al. (22) group 1	Naltrexone implant	6 months	1,000 mg
Krupitsky et al. (22) group 2	Oral treatment by naltrexone	6 months	50 mg naltrexone daily
Ruger et al. (29)	Oral treatment by naltrexone	24 months	50 mg daily
Sullivan et al. (34)	Injectable naltrexone administration	2 months	384 mg
Lee et al. (25)	Injectable extended release naltrexone	2 months	380 mg
Lee et al. (24)	Injectable extended release naltrexone	6 months	380 mg
Nunes et al. (28)	Injectable extended release naltrexone	6 months	380 mg

NR, not reported.

### Treatment outcomes

**Figure 2:**
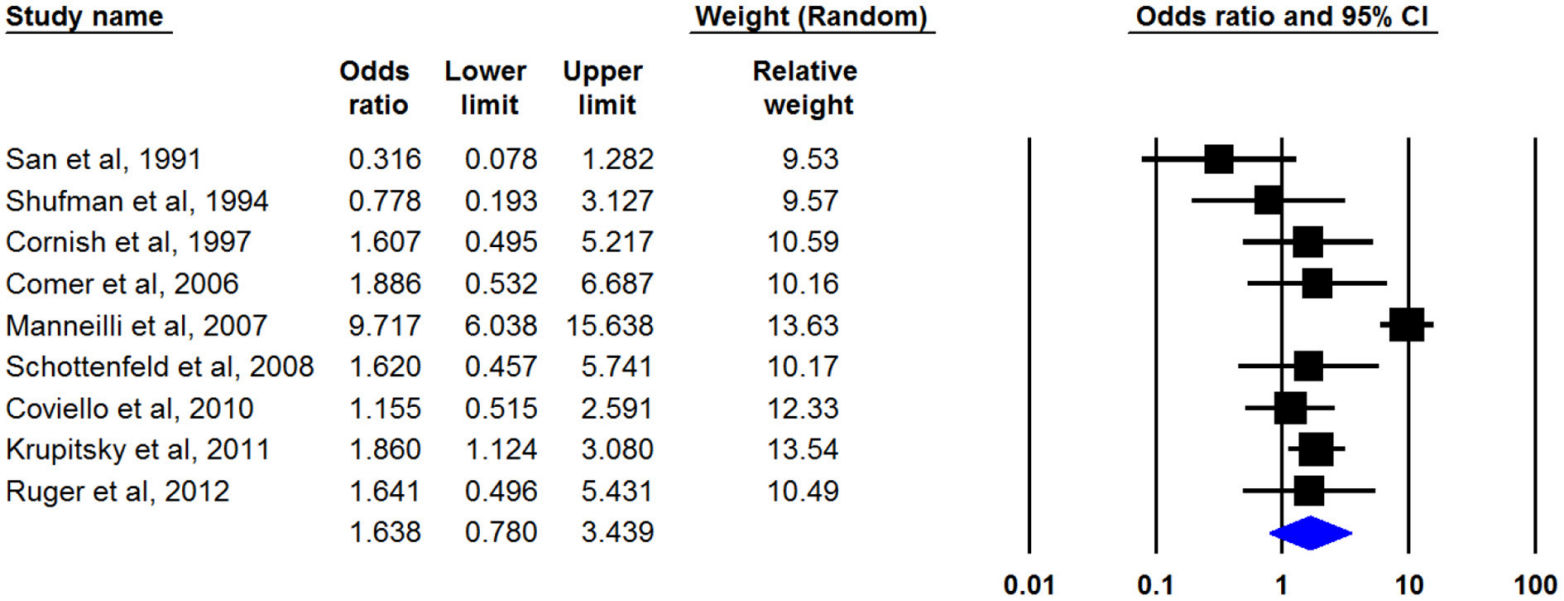
Pooled OR of retention in treatment analysis.

**Figure 3:**
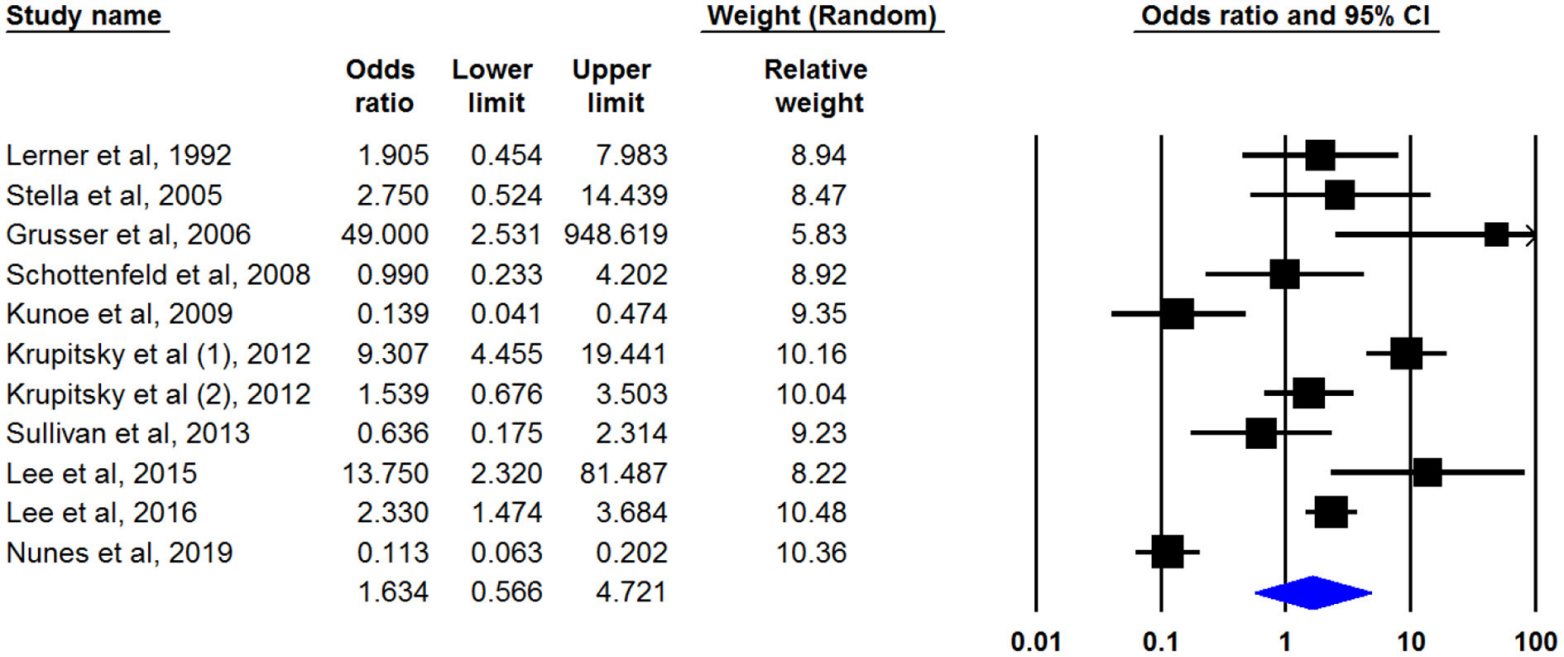
Pooled OR of being opioid free analysis.

Eight studies (17–19, 22, 27, 29, 30, 32) had defined treatment success as retention in treatment up to the last of the study and nine articles by (20, 21, 23–26, 28, 33, 34) had discussed the being opioid-free at the end of the study without any relapse as treatment outcome. Only one study had mentioned both outcomes (31). Our meta-analysis of both outcomes showed that naltrexone was useful for opioid dependency treatment whereas its effect was not statistically significant. The pooled OR of retention in treatment with naltrexone was 1.638 (95% CI, 0.780–3.439) (Figure 2). This number was 1.634 (95% CI, 0.566–4.721) for another outcome (being opioid-free without any relapse) (Figure 3). There was no evidence of publication bias in any of both outcomes (*p* > 0.05).

### Subgroup analysis

Both oral and injectable or implant naltrexone affected reducing opioid dependency in both outcomes but none of them were statistically significant except the effect of injectable naltrexone on retention in treatment with the pooled OR 1.864 (CI 95%, 1.167–2.977) (Table 4).

**Table 4:** Subgroup analysis for naltrexone type.

**tblheadOutcome**	**Type of naltrexone**	**No. of study**	**Pooler OR** **(95% CI)**	**Heterogeneity** ***I*^2^ (%)**	**Begg's test** ***p*-value**
Retention in treatment	Oral	7 studies	1.52 (0.54–4.24)	86.7	0.36
	Injectable or implant	2 studies	1.86 (1.16–2.97)	0.0	N/A
Being opioid free without relapse	Oral	4 studies	1.59 (0.87–2.90)	0.0	0.73
	Injectable or implant	7 studies	1.69 (0.35–8.03)	95.2	1.00

## Discussion

They included clinical trials that used a wide range of treatments with naltrexone between 3 weeks and 2 years. Naltrexone was associated with better efficacy in terms of retention in treatment and being opioid-free, whereas the findings were not significant. By route of naltrexone administration, injectable/implant naltrexone was more effective than oral ones for opioid dependency.

The systematic review by Jarvis et al. on 34 studies showed that extended-release naltrexone compared with placebo reduced opioid use, although its results could be confounded as it was mentioned by the author's (12). Additionally, a previous systematic review of 14 studies revealed non-statistically significant results for successful completion of treatment (OR = 0.78; 95% CI, 0.34–1.75) and use of opioids under treatment (OR = 0.85; 95% CI, 0.45–1.62) (35). A network meta-analysis conducted by Lim and colleagues which aimed to evaluate the effectiveness of different medication types for opioid-related disorders showed that the likelihood of treatment retention was statistically significantly higher for naltrexone than for controls [relative risk (RR) = 1.54; 95% CI, 1.26–1.90], and showed the average percent of treatment retention of 41.0% for naltrexone among all included studies (36). Furthermore, the RRs for the effectiveness of naltrexone compared with buprenorphine, methadone, and slow-release oral morphine were 0.72 (95% CI, 0.55–0.91), 0.59 (95% CI, 0.45–0.77), and 0.62 (95% CI, 0.38–0.99), respectively (36). Our study showed that naltrexone increased retention to treatment by 63.8% compared to the control group, which was not significant (95% CI, 0.78–3.44). The findings are almost by previously mentioned studies and the slight differences could be a result of different methodologies like the inclusion/exclusion criteria, search date, and methods for data synthesis. In this regard, the article by Timko et al. showed a wide range of rates of retention in treatment for opioid dependence from 3–88% at 3-months to 37–91% at 12-months of follow-up (37). Overall, naltrexone was the fourth most effective medication among medications for opioid-related disorders than controls in terms of treatment retention after methadone (RR = 2.62), and slow-release oral morphine (RR = 2.52), and buprenorphine (RR = 2.15) (36). The better efficacy of methadone for treatment retention was also shown in another study by (37). Therefore, the decision on the type of drug and route of administration is related to different factors like efficacy, safety, and accessibility.

Our results showed that naltrexone compared with controls increased the odds of being opioid-free by 1.63 times (95% CI, 0.57–4.72). In this regard, a systematic review and meta-analysis of 11 studies including 1,045 criminal-justice individuals showed that naltrexone significantly reduced the rate of reincarceration and opioid relapse by 30 and 37%, respectively, and it also improved opioid abstinence (RR = 1.38, 95% CI, 1.16–1.65) (38). The significant results of the study compared to our findings could be a result of restricting the inclusion criteria to only a certain population. To our findings, another systematic review of eight trials and 1,213 adult smokers showed no significant difference between naltrexone and placebo in long-term smoking abstinence (RR = 1.00; 95% CI, 0.66–1.51) (39). Some factors like being employed, a referral from private clinics, and daily heroin injection were associated with a longer retention period or willingness to treatment with naltrexone (40, 41). These factors should also be taken into consideration in treatment guidelines and planning for opioid cessation activities.

We found that injectable or implant naltrexone was associated with higher odds of retention in treatment (1.86 vs. 1.52) and being opioid-free without relapse (1.69 vs. 1.59) than oral naltrexone. Larney et al. conducted a meta-analysis on nine clinical trials and showed that both implants (RR = 0.57; 95% CI, 0.48–0.68) and oral naltrexone (RR: 0.57; 95% CI, 0.47–0.70) are associated with better outcomes than placebo in suppressing opioid use (42). A study that followed heroin-dependent people who were treated with oral or implant naltrexone showed that implant naltrexone was significantly associated with lower odds of opioid use than oral one (OR = 0.36; 95% CI, 0.16–0.82) (43). The results of a randomized controlled trial also showed that long-acting injectable naltrexone was associated with higher retention to treatment than oral one (hazard ratio = 2.18; 95% CI: 1.07–4.43) (44), which supports our findings. In addition, studies support the use of a combination of pharmacological and non-pharmacological studies for the treatment of opioid dependence. In this regard, a combination of naltrexone with behavioral therapy reduced the probability of reincarceration (OR = 0.30; 95% CI 0.12–0.76) (35).

The risk of bias assessment of the included articles showed that domains, including blinding of outcomes, followed by blinding of participants, and allocation concealment had the lowest quality among included articles. A similar study on naltrexone for opioid dependence showed the highest risk of bias in blinding of participants and outcome domains (12). Moreover, the quality assessment of the included articles of a systematic review that evaluated the effects of naltrexone on alcohol consumption showed a higher risk of bias in allocation concealment and incomplete outcomes (45). The findings are helpful for scientists to design further clinical trials considering the fact to have better blinding of outcomes and participants which would be helpful for evidence-based medicine.

To the best of our knowledge, it is one of the leading meta-analyses which evaluates the effects of naltrexone on the efficacy of treatment in patients with opioid use disorders. However, we acknowledge that the study has several limitations. Firstly, it is probable that we missed some relevant articles since we only searched two online databases, although we used a comprehensive approach for screening and search of gray literature. Secondly, despite using subgroup analysis to understand the source of heterogeneity, the effects of other potential sources of heterogeneity like the age of participants, country of residence, and duration of treatment on the efficacy of naltrexone have not been evaluated in the present study. Thirdly, different modalities were used in the control group which ranged from placebo to pharmacological (e.g., clonidine) and psychological interventions (e.g., counseling). It could lead to bias in pooling the estimates, so the findings should be interpreted with caution. Fourthly, all non-English articles were excluded that can lead to bias in the results. Moreover, both randomized and non-randomized clinical trials with different durations of treatment were included which can also lead to bias. Fifthly, the outcomes of the current study were limited to the efficacy measures and the safety of naltrexone on the patients with opioid dependence was not assessed. Sixthly, the duration of naltrexone treatment in the studies varied from 21 days to 24 months. This factor could influence on the results of the analysis.

## Conclusions

The study shows that naltrexone appears to be an effective treatment in terms of retention in treatment and being opioid-free, however, the findings were not significant. Also, the effects of the implant or injectable opioids were higher than oral ones. We recommend further large-scale observational studies and randomized controlled trials, as well as updated meta-analyses of those studies to evaluate their efficacies. Moreover, different types of treatment approaches and duration of treatment can be assessed in the next research.

## Data availability statement

The original contributions presented in the study are included in the article/supplementary material, further inquiries can be directed to the corresponding author/s.

## Author contributions

MS and MN designed the study. MZ analyzed the data and performed the statistical analyses. SG, SN, and MMZ drafted the initial manuscript. All authors reviewed the drafted manuscript for critical content and approved the final version of the manuscript.

## Funding

This study was supported by Shahid Beheshti University of Medical Sciences, Tehran, Iran.

## Conflict of interest

The authors declare that the research was conducted in the absence of any commercial or financial relationships that could be construed as a potential conflict of interest.

## Publisher's note

All claims expressed in this article are solely those of the authors and do not necessarily represent those of their affiliated organizations, or those of the publisher, the editors and the reviewers. Any product that may be evaluated in this article, or claim that may be made by its manufacturer, is not guaranteed or endorsed by the publisher.
